# Simplified Liquid Chromatography–Mass Spectrometry Methods for Gestagen Analysis in Animal Fat and Liver

**DOI:** 10.1021/acs.jafc.3c01200

**Published:** 2023-06-15

**Authors:** Randy W. Purves, Michelle West, Ratnadipsinh Vaghela, Jana Kinar, Yash Patel, Michael W. Belford, Bryn O. Shurmer

**Affiliations:** † Centre for Veterinary Drug Residues, Canadian Food Inspection Agency, Saskatoon, SK S7N 2R3, Canada; ‡ College of Pharmacy and Nutrition, University of Saskatchewan, Saskatoon, SK S7N 5E5, Canada; § Thermo Fisher Scientific, San Jose, California 95134, United States

**Keywords:** MRL, melengestrol acetate, MGA, mass spectrometry, LC−MS, food safety, progestogens

## Abstract

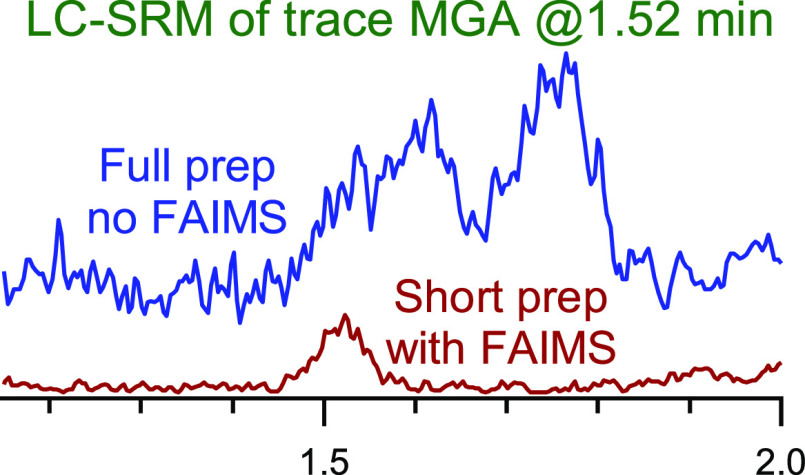

Gestagens, a class of veterinary drugs also called progestogens, are synthetic hormones used to increase feed efficiency and rate of gain in heifers. The Canadian Food Inspection Agency analyzes progestogens melengestrol acetate (MGA), megestrol acetate, and chlormadinone acetate using liquid chromatography–mass spectrometry (LC–MS). Our conventional gestagen method for kidney fat has many time-consuming steps, including solid-phase extraction. A sample preparation procedure having fewer clean-up steps was developed for routine diagnostic analysis of kidney fat and provided similar results faster, and at lower cost. A confirmatory liver method for gestagens, developed using salt-assisted extraction, employed minimal clean-up steps that resulted in high chemical background at the desired lower limit of quantification (LLOQ). Differential ion mobility spectrometry, specifically high-field asymmetric waveform ion mobility spectrometry (FAIMS), was used to filter chemical background in the gas phase. The effect of the ionization probe position on FAIMS parameters, including sensitivity, is described. With LC-FAIMS-MS, chemical background for each gestagen was virtually eliminated, resulting in a quantitative liver method having the desired 0.6 ng/g LLOQ and estimated limits of detection (LODs) up to 140 times lower than LC-MS. Incurred MGA samples, analyzed using kidney fat and liver methods from the same animal, show levels within the quantitative ranges of both methods.

## 1 Introduction

Veterinary drugs have an important role in animal health, but to ensure compliance and a safe food supply, the analysis of veterinary drug residues is required.^1^ Gestagens (progestogens) are a class of drugs that can be introduced into animal feed to increase feed efficiency and rate of gain in heifers.^2,3^ In particular, melengestrol acetate (MGA) is an effective oral progestational agent that is approved for use in some countries, including Canada and the USA, whereas in other jurisdictions such as the EU, the use of all gestagens is banned.^4^ A study in which heifers were fed radioactive MGA showed that fat tissues (including perirenal fat) contained more MGA than liver tissues,^5^ and therefore, fat is the preferred target tissue for MGA analysis.

Although gestagens have been analyzed in various matrices using several different techniques, such as gas chromatography–mass spectrometry,^6,7^ liquid chromatography–mass spectrometry (LC–MS) is preferred for gestagen analysis, because it offers a highly sensitive and selective platform without the need for derivitization.^8−14^ At the Canadian Food Inspection Agency (CFIA), the current LC–MS method for the analysis of gestagens in kidney fat was derived from an LC method that analyzes MGA, megestrol acetate (MA) and chlormadinone acetate (CMA).^15^ The gestagens MA and CMA are not approved for use, whereas the maximum residue limits (MRL) in Canada for MGA are 14 ng/g in fat and 6 ng/g in liver. Although the current methodology is effective in that it readily achieves the desired lower limit of quantification (LLOQ) of 5 ng/g, the sample preparation is time-consuming, taking ∼6.5 h. The method involves many clean-up steps that include solid-phase extraction (SPE) and the use of additional solvent. An extensive clean-up (particularly SPE) is used in all the aforementioned LC–MS methods for gestagen analysis in kidney fat. As our laboratory and others are constantly challenged to analyze more samples and more veterinary drug residues, simplified methods with less environmental impact are needed. Shortening this routinely used diagnostic method, without adversely affecting analytical parameters, would result in significant time savings.

The CFIA was recently tasked with developing a confirmatory (non-routine) method for the analysis of gestagens in liver, and this provided an opportunity to revisit the gestagen sample preparation protocol, especially since LC–MS instrumentation is much more sensitive and selective than even a decade ago. In addition, the advancement of complementary techniques, such as ion mobility, can also be used to improve the separation capacity of the analysis. High-field asymmetric waveform ion mobility spectrometry (FAIMS) is a type of differential ion mobility that is placed after the ionization source and before the mass spectrometer orifice. Since gas-phase ion separation in FAIMS is based on changes in ion mobility, FAIMS provides additional selectivity that can help minimize chemical background in LC–MS analysis.^16−18^ FAIMS has been used in different applications, including quantification of small molecules.^19−22^ The development of an improved interface region,^23,24^ integral to a second-generation FAIMS (i.e., FAIMS Pro), has provided a robust interface for diagnostic veterinary drug residue analysis.^25^ For thyreostats, an LC-FAIMS-MS diagnostic method showed significant chemical background reduction resulting in improvements in the LLOQ of up to 10×.^25^ In addition, since interface turbulence has been minimized in the new FAIMS, the ionization probe tip can now be placed closer to the FAIMS inlet compared with the first-generation FAIMS. Although ions may not be fully desolvated,^26^ by sampling more of the electrospray plume, sensitivity should be improved.

We hypothesized that instead of using time-consuming clean-up steps during sample preparation, shorter, simpler methods to analyze kidney fat and/or liver samples could be used while relying more upon the capabilities of mass spectrometry and its related techniques.^27^ In particular, if the simplified sample preparation resulted in an analysis that produced excessive chemical background interference, FAIMS would be used to clean up the samples in the gas phase (after ionization and before entering the mass spectrometer), thereby giving the required sensitivity and selectivity needed to achieve desired levels of quantification.

## 2 Materials and Methods

### 2.1 Tissue Material

Bovine kidney fat and liver samples that had previously been analyzed for progestogens (gestagens) were provided by the Centre for Veterinary Drug Residues at the CFIA (Saskatoon, SK, Canada). All samples were stored at −20 °C prior to extraction.

### 2.2 Chemicals and Reagents

Ethyl acetate, methanol, and hexane were obtained from Caledon Laboratory Chemicals (Georgetown, ON, Canada). Acetonitrile, magnesium chloride hexahydrate, magnesium sulfate, sodium sulfate, sodium chloride, methanol, formic acid, and sodium hydroxide were obtained from Fisher Scientific (Ottawa, ON, Canada). Hydrochloric acid was obtained from VWR (Mississauga, ON, Canada). Water used was purified by reverse osmosis followed by deionization, adsorption, and filtration.

Reference materials of CMA, MA, MGA, and MGA-d_3_ were obtained from Sigma-Aldrich (Oakville, ON, Canada). All stock and working solutions of these reference materials were prepared in methanol and stored at 4 °C, protected from light.

### 2.3 Calibration Curves and Quality Control (QC) Samples

#### 2.3.1 Chemical Standard Calibration Curves and Matrix-Fortified QCs in Kidney Fat

For both the full and shortened methods for the analysis of bovine kidney fat, a chemical standard calibration curve was prepared by diluting a 1.0 μg/mL working solution having all three analytes with 70:30 acetonitrile/water containing 0.2% aqueous hydrochloric acid to obtain tissue equivalent concentrations of 5, 10, 20, and 40 ng/g. Matrix-fortified controls were also prepared at 5, 10, and 20 ng/g and used for QC purposes. For the internal standard, 25 μL of a 1.0 μg/mL working solution of MGA-d_3_ was added to all extraction tubes to give a tissue equivalent concentration of 12.5 ng/g.

#### 2.3.2 Matrix-Fortified Calibration Curves in Liver

For the analysis of bovine liver, a matrix-fortified calibration curve of 0.6, 3.0, 6.0, 9.0, and 12.0 ng/g was prepared. For 0.6 ng/g, 12 μL of a 0.1 μg/mL working standard was spiked onto a 2 g portion of liver. For the remaining four points, 6, 12, 18, and 24 μL of a 1.0 μg/mL working standard were spiked onto separate 2 g portions of liver. A separate matrix-fortified control was prepared at 6.0 ng/g and used for the QC. All spikes were allowed to rest for at least 15 min prior to beginning the extraction procedure. MGA-d_3_ (15 μL of a 1.0 μg/mL working standard) was added to all extraction tubes for use as an internal standard.

### 2.4 Extraction Methods

#### 2.4.1 Full Kidney Fat Analysis

Our original method, which will be referred to as the “full method”, was developed in-house.^15^ To render kidney fat tissue, ∼25 g of adipose tissue, cut into ∼2 cm cubes, was placed in a glass funnel to which silanized glass wool was packed into the bottom. The funnel was placed over a 250 mL beaker containing a 20 mL scintillation vial and the beaker was placed into a microwave oven. The kidney fat was microwaved at a power setting of 50% for approximately 1.5–2 min. If needed, the sample was microwaved for additional 20–30 s intervals with a power setting of 30–40% until the kidney fat started to melt and drip through the funnel into the scintillation vial. Samples and blank matrix controls were prepared by weighing 2 ± 0.02 g of the microwave-rendered bovine kidney fat into 50 mL polypropylene centrifuge tubes. Once the tubes were fortified with the working solution and internal standard, as necessary, the progestogens were extracted from the kidney fat with 5 mL of acetonitrile by heating the tubes in a water bath set at 60 °C for 5 min until the rendered kidney fat had liquefied. This was followed by shaking the tubes for 3 min and centrifuging for 7 min at 1160 × *g* with the temperature set to −5 °C. The supernatant was decanted into a 15 mL glass centrifuge tube. A second volume of 5 mL of acetonitrile was added to the 50 mL polypropylene centrifuge tubes and again these tubes were heated in the water bath to liquefy the kidney fat, then shaken, and centrifuged. The supernatant from the second extraction was combined with the supernatant from the first extraction in the 15 mL glass centrifuge tube.

A volume of 2 mL of hexane was added to the combined supernatants followed by shaking for 1 min and centrifuging for 7 min at 1160 × *g* with the temperature set to −5 °C. After removing the hexane layer to waste, the hexane wash was repeated, again removing the hexane layer to waste. The remaining extract was evaporated to dryness using a nitrogen evaporator (N-Evap, Organomation, Berlin, MA, USA) with a water bath set to 60 °C. The sample extracts were reconstituted with 4 mL of hexane, and lipids were saponified using 1 mL of 0.1 M aqueous sodium hydroxide, 0.5 mL of 1 M aqueous magnesium chloride, and vortex mixing. After heating in a water bath set at 60 °C for 15 min, the samples were centrifuged for 5 min at 1160 × *g* with the temperature set to −5 °C. The hexane supernatant was transferred to a new 15 mL glass tube, and 4 mL of hexane only was added to the 15 mL glass centrifuge tube. After again heating in a water bath at 60 °C for 15 min and centrifuging for 5 min at 1160 × *g* (temperature set to −5 °C), the hexane supernatant was collected and combined with the first hexane supernatant in the 15 mL glass tube. The combined hexane was evaporated to dryness using an N-Evap with the temperature set to 60 °C. The extract was reconstituted with 1 mL of hexane and loaded onto a cyanopropyl-endcapped (CN-E) 3 mL, 500 mg packing SPE cartridge (Waters Limited, Mississauga, ON, Canada) that had been conditioned with 5 mL of ethyl acetate and 6 mL of hexane. The glass tube was washed two times with 1 mL of hexane, each time the hexane wash was added to the SPE cartridge. The SPE cartridge was then washed with 5 mL of hexane and dried under full vacuum for 2 min. The progestogens were eluted from the cartridge using 6 mL of 20% ethyl acetate in hexane into a 15 mL glass centrifuge tube.

The extract was evaporated to dryness using the N-Evap with the temperature set to 60 °C and then reconstituted into 1 mL of 70:30 acetonitrile/water. After allowing the samples to sit for 15 min, 10 μL of 0.2% aqueous hydrochloric acid was added to each tube. Each sample was vortexed and filtered into a 2 mL LC vial using a 13 mm Acrodisc syringe filter [0.2 μm poly(tetrafluoroethylene) (PTFE)] prior to analysis.

#### 2.4.2 Shortened Kidney Fat Analysis

The shortened analysis uses the same procedure as the full method above *except* the entire second paragraph in Section 2.4.1 is omitted. That is, after combining the acetonitrile extracts in the 15 mL glass centrifugation tube at the end of paragraph 1, this combined extract is evaporated to dryness using a nitrogen evaporator with the temperature set to 60 °C as per the first sentence in paragraph 3. The rest of paragraph 3 (reconstitution, adding acid, vortex, and filtering) was carried out using the same procedure. Thus, the shortened method skips the hexane de-fatting and SPE steps of the full method.

#### 2.4.3 Liver Analysis

Samples and blank matrix controls were prepared by weighing 2 ± 0.02 g of bovine liver into 50 mL polypropylene centrifuge tubes. Once the tubes were fortified with the working solution and internal standard, as necessary, 5.0 mL of acetonitrile was added and the tissue was homogenized with a polytron mixer until well blended. After shaking for 2 min on a horizontal shaker, 1.2 g of NaCl was added and the samples were shaken for another 2 min. Next, 4.0 g of Na_2_SO_4_ and 0.50 g of MgSO_4_ were added, and the samples were shaken for a final 2 min at a high speed. Samples were shaken by hand as necessary to bring the tissue into solution if the mechanical shaking was incomplete. The samples were centrifuged at ∼6100 × *g* and at room temperature for 30 min. A 500 μL portion of the sample extract was transferred into a syringeless filter vial (Mini-UniPrep PTFE filter vial, 0.2 μm, Whatman). Extracts were evaporated to dryness with an N-Evap at room temperature and reconstituted with 250 μL of 70:30 acetonitrile/0.1% aqueous formic acid.

### 2.5 Analysis Using LC-Selective Reaction Monitoring (SRM) and LC-FAIMS-SRM

A Vanquish ultra-high performance liquid chromatographic system was coupled to a Thermo Scientific TSQ Altis Plus triple quadrupole mass spectrometer, which was equipped with a FAIMS Pro interface. The column was a Waters BEH C18 (2.1 × 50 mm, 1.7 μm); the column temperature was set to 45 °C, and the autosampler was set to 5 °C. The mobile phases and gradient (liquid flow rate of 400 μL/min) are given in Table 1.

**Table 1:** Liquid Chromatography (LC) Gradient and Mass Spectrometry Conditions for the Quantification of MGA, MA, and CMA^a^

LC Gradient		
time	% A	% B
0	35	65
0.2	35	65
2.2	25	75
2.3	1	99
3.5	1	99
3.6	35	65
5	35	65
		

SRM Transitions		
gestagen	transition	CE
MGA	397 → 337	14 (Q)
	397 → 279	20
MA	385 → 267	19 (Q)
	385 → 325	25
CMA	405 → 309	16 (Q)
	405 → 345	12
MGA-d_3_	400 → 337	14

a Note: A = 0.1% formic acid (aq) and B = methanol; (Q) transition used for quantification.

The Thermo TSQ Altis plus mass spectrometer used heated electrospray ionization (HESI) in positive ion mode (3300 V) with an ion transfer tube temperature of 325 °C and a desolvation temperature of 350 °C. The sheath gas and aux gas flows were set to 60 and 15, respectively. The SRM conditions are also given in Table 1.

When the FAIMS was used, the LC and MS conditions remained the same. FAIMS compensation voltage (CV) optimization was carried out using the “scan CV” feature in the Tune software. For the HESI probe optimization study, 13 CV values were cycled in 1 V steps. The Tune software window was also used to set both electrode temperatures to 80 °C in User Defined FAIMS Mode and the total carrier gas flow rate was 5.6 L/min of nitrogen. The HESI probe position was optimized and set to vertical position LM, which is between L and M, with the probe positioned just beyond the 1 mark, at approximately 1.3 on the source housing. This same ionization probe position was used in both LC-SRM and LC-FAIMS-SRM experiments.

### 2.6 Data Analysis

The quantification of the data was carried out using Thermo Scientific TraceFinder 5.1 software. For each gestagen, the ratio of the area of that gestagen/MGA-d_3_ internal standard area was calculated for each point used in the calibration curve. A linear regression curve with 1/X weighting was used for quantification.

Igor Pro 6.3 (Wavemetrics, Lake Oswego, OR, USA) technical graphing and data analysis software was used to create all figures except Figure 7 (Excel). Raw data was first converted into text format, before being loaded into the software as *x*,*y* waves, which enabled flexibility in data presentation.

## 3 Results and Discussion

### 3.1 Comparison of Shortened and Full Kidney Fat Methods

**Figure 1:**
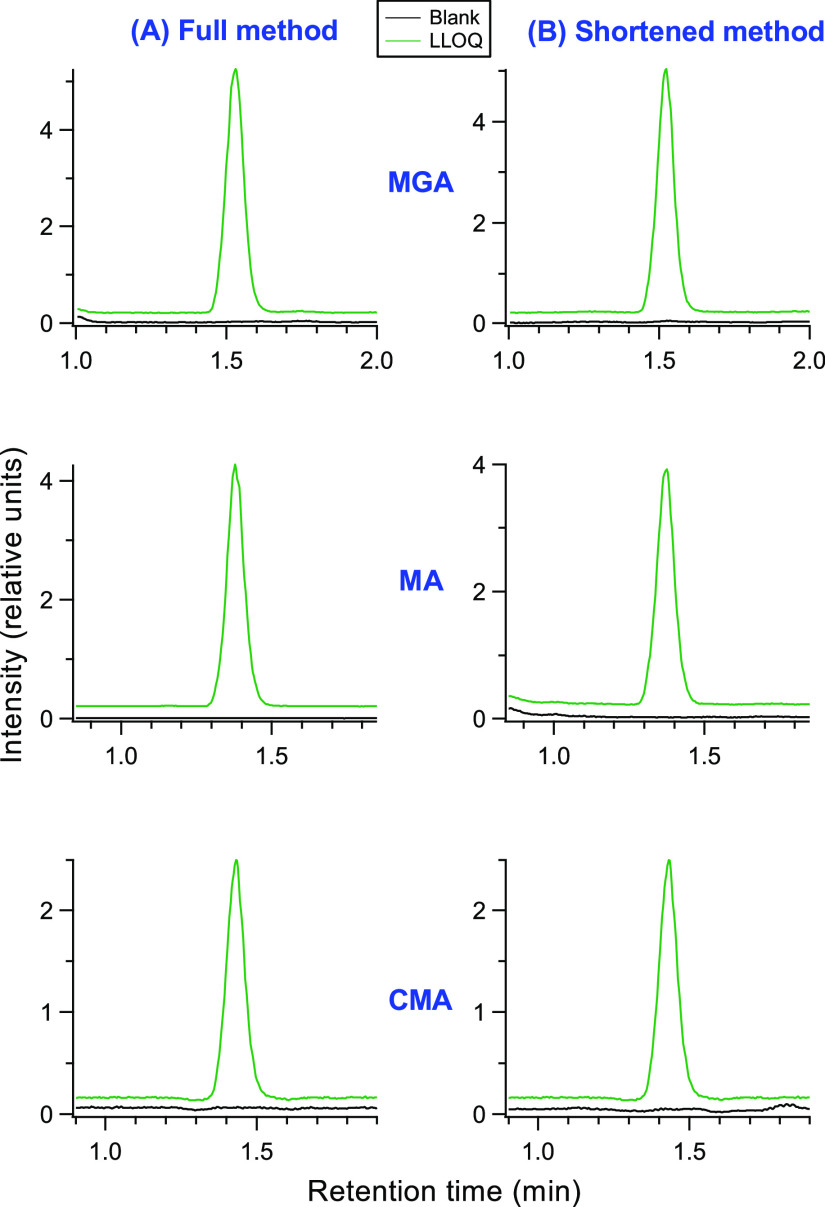
Comparison of the quantitative transitions for three gestagens (MGA, MA, and CMA) in the matrix blank and LLOQ (5 ng/g) samples using (A) full and (B) shortened kidney fat methods. The LLOQ traces are offset by 0.2 units in MGA and MA, and 0.1 units in CMA.

Figure 1 shows a comparison of the LC-SRM transitions used for quantification of the three gestagens using the full (Figure 1A) and shortened (Figure 1B) methods for kidney fat using LC-SRM. The figure shows a matrix blank and a fortified kidney fat sample containing 5 ng/g for each gestagen using the full and shortened methods. For all traces, the 5 ng/g sample is offset by ∼5% of full scale for clarity. The value of 5 ng/g represents the LLOQ and is approximately 1/3 of the MRL of 14 ng/g. Figure 1A shows that although the full clean-up is time-consuming, the 5 ng/g sample is readily distinguished from the matrix blank. The simplified clean-up in Figure 1B does lead to some additional background, most notably for MA and CMA at the beginning and end of the LC-SRM traces, respectively. Fortunately, this background, which is well below the signal for the 5 ng/g sample, is far enough removed from the retention time of MA and CMA that they are still easily quantified. The peaks areas for the gestagens are comparable between the methods, and overall, both methods are suitable for the analysis of the gestagens (as will be discussed further in Section 3.4). However, since the sample preparation takes approximately half the time, and the use of SPE is eliminated, the simplified gestagen method is preferred and has been verified and implemented as our routine diagnostic analysis method.

#### 3.1.1 Key Validation Parameters for the Shortened Kidney Fat Method

Method validation target criteria were based on Codex Alimentarius guidelines CAC/GL 71-2009, and all method criteria passed. Method validation parameters for the shortened quantitative method were derived from 9 months of sample analysis (unless otherwise indicated). A linear chemical standard calibration curve is used with internal standard correction to compensate for possible recovery differences in matrix. Selectivity showed no false negatives were greater than the LLOQ, and no false positives were observed for the three gestagens (*n* = 100 for MA and CMA, *n* = 103 for MGA). Absolute recovery was calculated by comparing the average of matrix-matched and matrix-fortified spikes at 5.0 and 20.0 ng/g, respectively. Absolute recovery for MA is 100%, for CMA is 95%, and for MGA is 87%. For the analytical range (5–40 ng/g), back-calculated recoveries for matrix-fortified spikes were averaged utilizing MGA-d_3_ as the internal standard for MA, CMA, and MGA, and the % recoveries are shown in Table S1. Within-run precision was determined using a relative response factor for all chemical calibration standards and matrix-fortified spikes; the within-run repeatability was less than 18% across the quantitative range, as well as at 5 ng/g for all runs. Intermediate precision was determined using the standard deviation (SD) for replicate measurements of 5.0, 10.0, and 20.0 ng/g matrix spikes, and the values are also shown in Table S1. Trueness was determined as the average trueness/bias observed in 13 data points at 10.0 ng/g, which is ±17%, 19%, and 6% for MA, CMA, and MGA, respectively.

Since the desired LLOQ of 5 ng/g is readily achieved, the limit of detection (LOD) was estimated based on matrix blanks using the mean response of the matrix blank + 3 × SD. The LODs were estimated for both the full kidney fat method and the shortened kidney fat method for both the quantitative and qualifier transitions for each gestagen, with the highest LOD being chosen from the two transitions. The chemical background levels were somewhat higher using the shortened kidney fat method resulting in estimated LOD values for MGA, MA, and CMA of 0.11, 0.048, and 0.17 ng/g, respectively, versus 0.035, 0.020, and 0.15 ng/g, respectively, for the full kidney fat method. Although the LODs are slightly higher in the shortened method, they are still well below the desired 5 ng/g LLOQ as illustrated in Figure 1.

### 3.2 Bovine Liver Analysis and the Use of LC-FAIMS-MS

**Figure 2:**
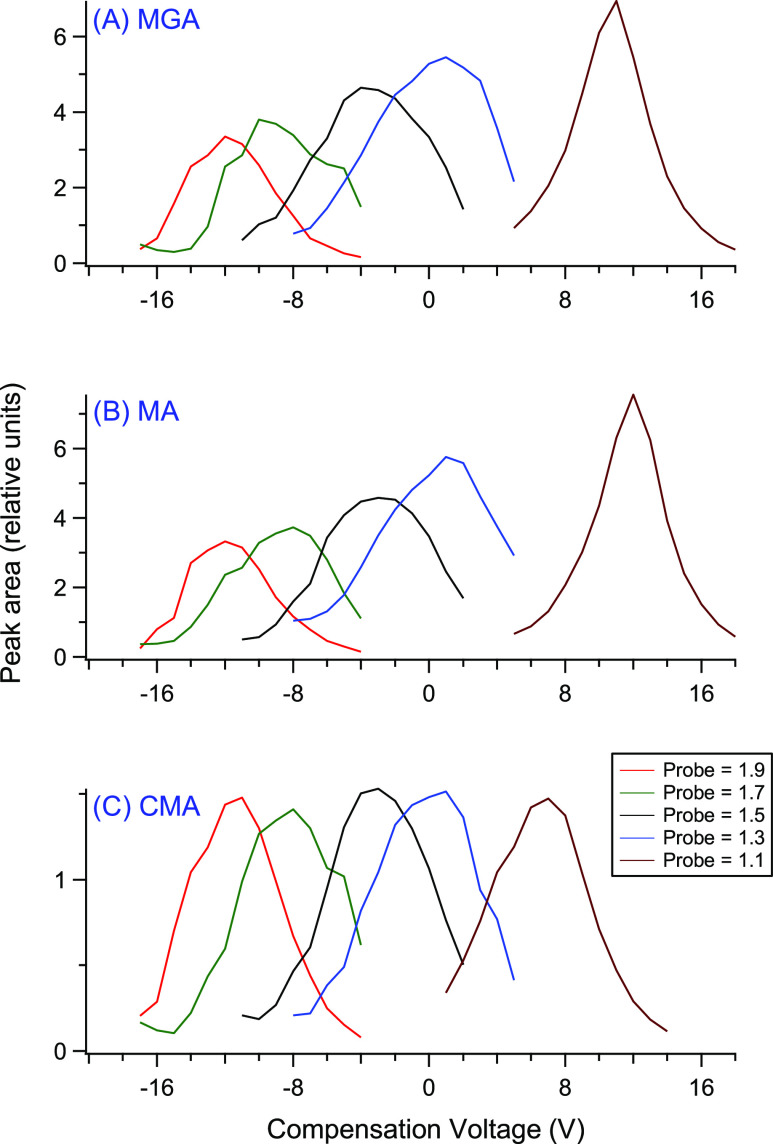
Effect of the HESI probe position on the optimum CV transmission value and the peak area for (A) melengestrol acetate (MGA), (B) megestrol acetate (MA), and (C) chlormadinone acetate (CMA). A HESI probe position of 1.3 was used for acquiring quantitative data.

Historically, with diagnostic methods, we aimed to have an LLOQ of at least 0.5 × MRL. However, recent legislative changes in the European Union require performance data to be collected at a level of 0.1 × MRL as per EU Regulation 2021/808 for authorized substances. Therefore, for newly established methods, which includes gestagen analysis in bovine liver, we targeted an LLOQ of 0.6 ng/g. Similar to kidney fat, with bovine liver we implemented a simplified sample preparation protocol, which did not use SPE and clean-up steps, as described in Section 2.4.3. Using the liver method, at the desired LLOQ, which was almost 10 × lower compared with the kidney fat method, significantly more chemical background was observed. A longer chromatographic run was attempted to help reduce interferences, but there was still significant chemical background interference in the region of analyte elution for the three gestagens (not shown). Since extra selectivity was needed, LC-FAIMS-MS was employed in the analysis of bovine liver. Typically, with FAIMS, the HESI probe is placed at a distance where complete desolvation occurs,^26^ which includes the LC-FAIMS-MS diagnostic method for thyreostats.^25^ However, the HESI probe can now be moved closer to the FAIMS Pro inlet without robustness issues, enabling more of the electrospray plume to be sampled, which can lead to improved signal intensity. Figure 2 shows peak area as a function of CV for the three gestagens at five different HESI probe positions. Separate injections at each probe position cycled through 13 CV values (as described in Section 2.5). At a HESI probe distance of 1.9 from the FAIMS inlet, or further away (higher numbers), the optimum CV value for maximizing peak area remains constant, suggesting complete desolvation is taking place. However, as the probe is moved closer to the FAIMS inlet (lower numbers), as is shown in Figure 2, the optimal CV values for peak area shift to less negative values, and the peak areas change. The shift in the optimum CV is believed to be a result of a clustering/declustering mechanism between the analyte ions and solvent molecules (in this case methanol) that has been described previously.^28,29^ At a HESI probe position of 1.7, there is only a slight shift in CV suggesting minimal solvation accompanied by a slight increase in the peak areas of two of the ions. As the HESI probe is moved closer to 1.5 and then 1.3, the shift in the optimum CV from the desolvated condition becomes greater. Finally, moving the HESI probe position to 1.1 results in a large change in the optimum CV, suggesting that a significant amount of solvent vapor is entering the FAIMS at this probe position. Interestingly, at this position the optimal CV value for CMA begins to deviate markedly from MGA or MA, illustrating the power of using gas-phase additives (vapor from the LC solvent in this instance) to improve FAIMS separation capabilities.^26,29^ Without solvation effects, the intensity is expected to increase as the probe moves closer because more of the HESI plume is sampled. However, since the solvent effects are ion dependent in FAIMS,^24^ the effects can vary among ions as the HESI probe is moved closer. In this study, two of the analytes experienced an increase in their optimum peak areas, whereas the peak area of CMA remained approximately the same. In general, we find for our high liquid flow rate LC-SRM methods that higher peak areas are obtained as the HESI probe moves closer. However, at HESI probe positions closer than 1.3, robustness usually decreases, and therefore, we typically use a distance of 1.3 as it gives the best trade-off between sensitivity and robustness. For LC-FAIMS-SRM, sensitivity of two analytes increased as the HESI probe position was moved closer, but like LC-SRM, in order to ensure good robustness, a value of 1.3 was also selected. Although gas additives can be added directly to FAIMS,^26^ since this addition is not a standard feature of the FAIMS Pro, the use of the HESI probe position offers a means to use gas modifiers without instrument modifications. However, unlike adding gas additives directly into the FAIMS gas flow,^26^ this approach has limitations. The gas modifier must be the same as the LC solvent, and if the gradient is altered, the optimum CV value could change in response to a different percent composition of the solvent from the gradient. Moving the probe position or changing temperatures or gas flow rates could also potentially result in a change in optimal CV. Thus, when using probe positions that do not produce completely desolvated ions, the CV values should be checked (i.e., “scan CV” function in Tune) before proceeding with an analysis, and variables held constant throughout the analysis. Our practice when carrying out diagnostic analyses using LC-FAIMS-SRM is to confirm the CV values using our system suitability run at the beginning of the analysis, which utilizes the “scan CV” function, and then run the same sample again at the end of the analysis using the “scan CV” function. We have observed if experimental conditions are not changed, the optimum CV value will remain stable. This is supported by Figure S1 that shows peak areas for the three analytes using LC-FAIMS-SRM with a HESI probe at position 1.3 for 175 injections of a 6 ng/g matrix-fortified standard (∼18 h). The relative standard deviation values in this figure were <5% for all three analytes.

**Figure 3:**
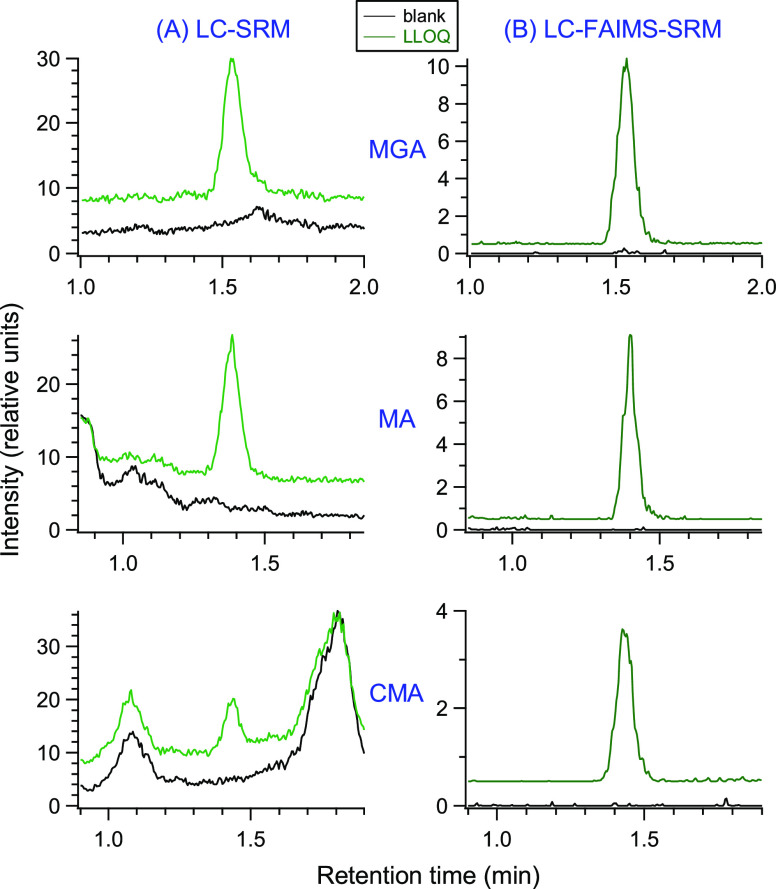
Comparison of the quantitative transitions for three gestagens (MGA, MA, and CMA) in the matrix blank and LLOQ (0.6 ng/g) samples using the new liver method and analyzing with (A) LC-SRM and (B) LC-FAIMS-SRM. The LLOQ traces are offset by 5 units in LC-SRM and 0.5 units in LC-FAIMS-SRM.

With the optimal FAIMS conditions, including the use of probe position 1.3, bovine liver samples were analyzed using LC-FAIMS-SRM. Figure 3 shows traces for the quantitative transitions for both the matrix blank and the LLOQ (0.6 ng/g) sample for each of the three gestagens using the new liver method with (A) LC-SRM and (B) LC-FAIMS-SRM. Compared to the desired LLOQ level of 0.6 ng/g, the LC-SRM matrix blank traces have a significant chemical background, which will adversely affect quantification and detection limits. To estimate the LOD values, again the mean response of the matrix blank + 3 × SD was calculated for both the quantitative and qualifier transitions with the highest LOD being chosen from the two transitions. For MGA, MA, and CMA, the estimated LOD values were 0.15, 0.14, and 0.25 ng/g, respectively. Figure 3 shows that with the optimized LC-FAIMS-SRM conditions, the chemical background noise has been virtually eliminated for all three gestagens, with background levels at or near zero in the matrix blanks. Thus, LOD estimates with LC-FAIMS-SRM are much lower at 0.020, 0.0010, and 0.0050 ng/g for MGA, MA, and CMA, respectively, which are 7.5–140 times lower than LC-SRM. As the figure shows, although the background drastically improves with FAIMS, this is accompanied by some loss in the total signal intensity. For the three quantitative traces shown in Figure 3, when comparing peak areas of LC-FAIMS-SRM to LC-SRM, the peak areas are 46% for MGA, 47% for MA, and 30% for MGA. When measuring signal-to-noise (S/N) as (peak intensity – mean response of matrix background)/SD of matrix background, the S/N values in the figure for the LLOQ samples of MGA, MA, and CMA are 40, 34, and 22, respectively, for LC-SRM versus 117, 2100, and 1600, respectively, for LC-FAIMS-SRM.

### 3.3 Incurred MGA Samples Analyzed in Kidney Fat and Liver

**Figure 4:**
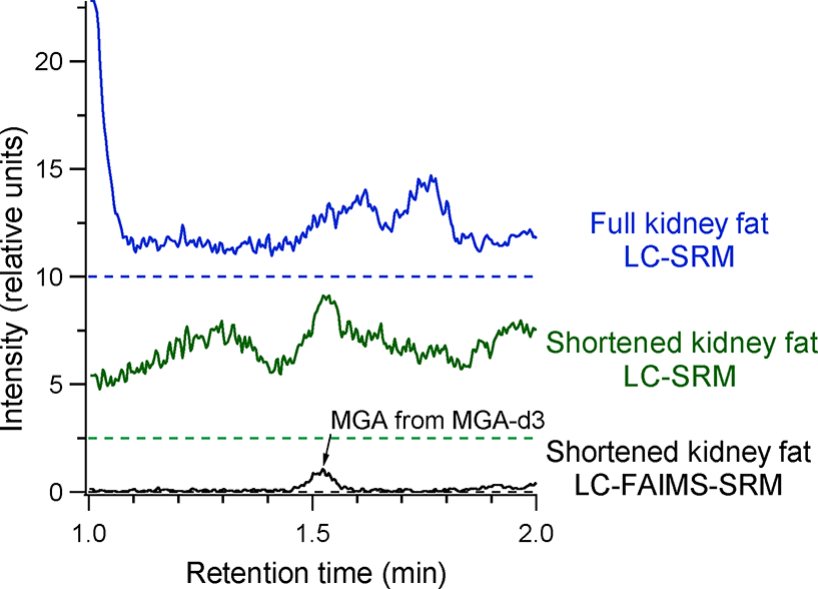
Comparison of the matrix blank samples for kidney fat analysis using the full kidney fat method and the shortened kidney fat method (both with LC-SRM), and the shortened kidney fat method with LC-FAIMS-SRM. The dashed lines represent the baselines for the respective analysis. The MGA contribution from the MGA-d_3_ internal standard is clearly visible using LC-FAIMS-SRM.

Since the initiation of the development of this confirmatory method in liver, for any diagnostic samples found to have an incurred amount of MGA in bovine kidney fat, the corresponding liver sample from the same animal was saved, where possible. These samples enabled a comparison of the MGA amounts in kidney fat and liver to be made, as well as a comparison among the different methods. Although the use of FAIMS is not required in the analysis of kidney fat, for comparative purposes, LC-FAIMS-SRM was also used to analyze kidney fat. A closer examination of the matrix blanks showed an interesting observation. Figure 4 shows the matrix blanks for the analysis of MGA using the full kidney fat and shortened kidney fat methods with LC-SRM, and the shortened kidney fat method with LC-FAIMS-SRM. The colored dashed lines in the figure indicate the baselines for the respective methods. The shortened kidney fat method has intense chemical background as the baseline is elevated throughout the analysis. The trace suggests the presence of an MGA peak, but it is partially obscured by the chemical background and the S/N < 3. The full kidney fat method has less background in general, but background in the area of MGA obscures the peak. It is only when LC-FAIMS-SRM is used that a distinct peak is observed for MGA in the matrix blank (S/N ∼ 40). With LC-FAIMS-SRM, the peak area of MGA in the matrix blank is about 0.5% the peak area of the 5 ng/g sample. This peak is consistently observed in the kidney fat matrix blank (i.e., no analyte, only internal standard added) using LC-FAIMS-SRM but was not observed in an experiment using a double matrix blank (i.e., no analyte or internal standard added). This peak was confirmed to be the result of a small MGA contamination arising from the use of MGA-d_3_ by QA acceptance testing of the MGA-d_3_ standard. This contaminant was only clearly observed because of the greatly improved S/N with FAIMS. Note that the same internal standard is being used for the liver method, and the presence of this contaminant peak is what limits the LOD and S/N values for MGA discussed in Section 3.2.

**Figure 5:**
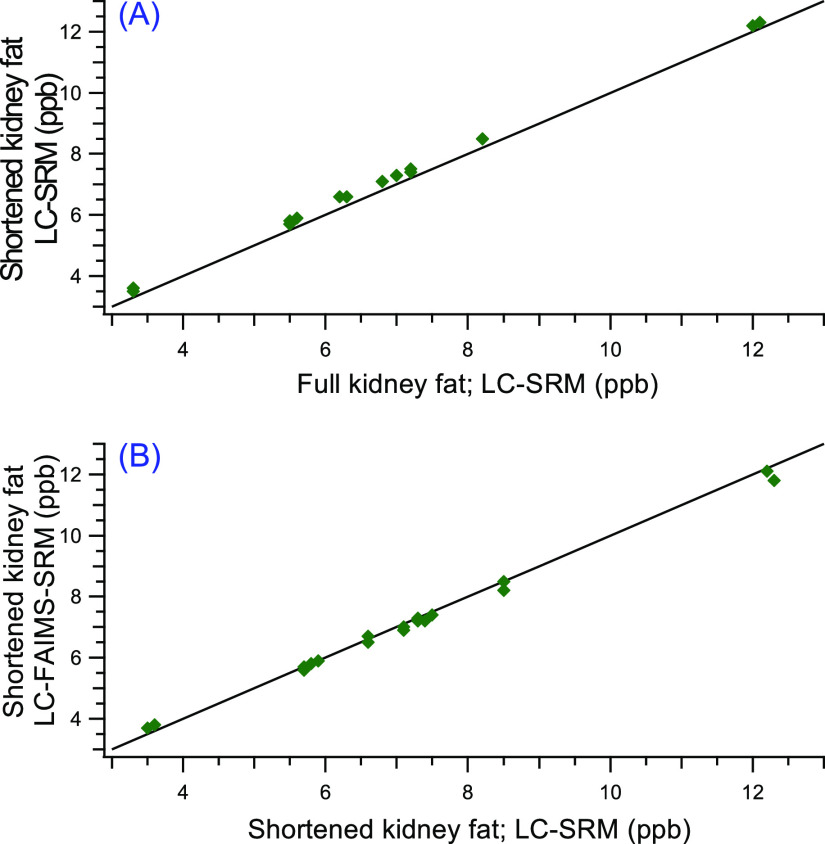
Using nine incurred samples for MGA in kidney fat (run in duplicate), (A) comparison of the full and shortened kidney fat methods using LC-SRM and (B) comparison of the shortened kidney fat method using LC-SRM and LC-FAIMS-SRM.

A comparison of methods for the analysis of kidney fat is shown in Figure 5A by plotting the incurred sample results from the original full kidney fat method versus the shortened kidney fat method. Ideally, this comparison would be carried out over multiple analyses and would contain more data points, however, as there are only nine incurred samples, this comparison is preliminary. Nonetheless, using this approach, all the points will fall on the 45° straight line in the plot if the methods are equivalent. Figure 5B used the shortened kidney fat method and shows a comparison of the results for LC-SRM and LC-FAIMS-SRM for the incurred MGA samples. In addition to the results for the incurred samples shown in Figure 5A,B, quantitative method performance results for the three gestagens using the three different methods for analysis of kidney fat are shown in Table S2.

**Figure 6:**
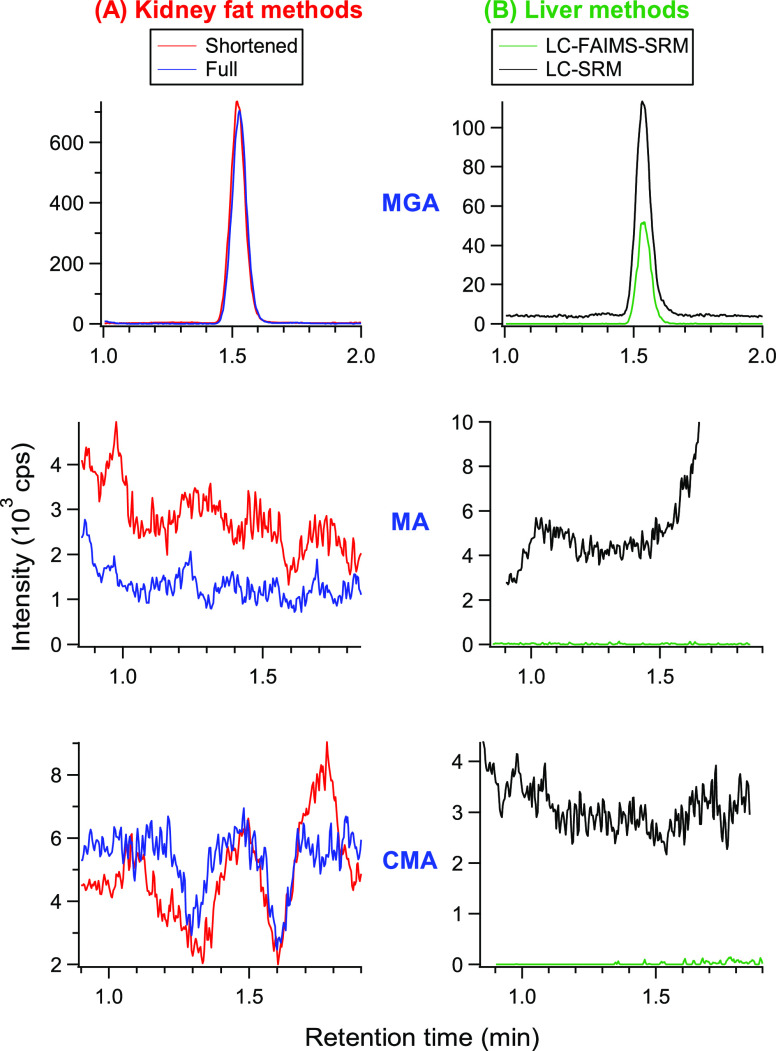
Quantitative transitions for MGA, MA, and CMA using tissues from an animal (A3) having incurred MGA. (A) Results obtained using the kidney fat methods. (B) Results for the corresponding liver sample using LC-SRM and LC-FAIMS-SRM.

When heifers had kidney fat samples test positive for MGA, their liver samples were also analyzed. Figure 6 shows the quantitative traces from an analysis of both kidney fat and liver from animal A3. Figure 6A shows results from kidney fat using both the full and shortened methods, whereas Figure 6B shows results from liver samples prepared using the new liver method and analyzed with both LC-SRM and LC-FAIMS-SRM. In Figure 6A, the traces are similar, illustrating the applicability of either method for analyzing kidney fat samples. Figure 6B illustrates that despite the ∼50% drop in signal intensity of MGA, the S/N is significantly improved due to the very large reduction of the chemical background, which gives large improvements in LODs as is discussed in Section 3.2.

**Figure 7:**
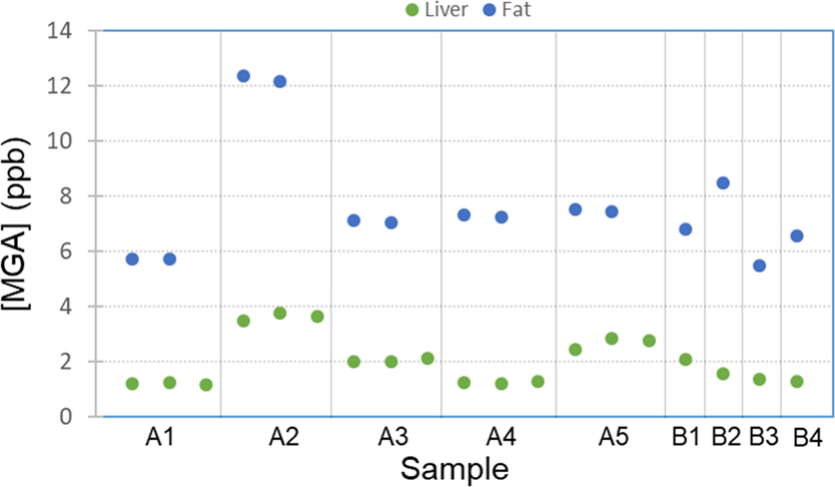
Concentration of MGA for incurred samples measured using the new liver method with LC-FAIMS-SRM (liver) and the shortened kidney fat method with LC-SRM (fat). Samples A1–A5 were acquired during method development, whereas samples B1–B4 were acquired during screening since the methods were implemented. Values are given in Table S4.

Results for incurred samples of MGA are shown in Figure 7. For five samples acquired during method development (A1-A5), the liver tissue results (run in triplicate) are presented along with the kidney fat tissue results (run in duplicate) from the same animal. The quantitative method performance results for the new liver method with LC-FAIMS-SRM, used to determine the values for the gestagens in animals A1–A5 in Figure 7, are shown in Table S3. The % recoveries for all three analytes in Table S3 are between 93 and 107%. Figure 7 shows that when MGA is quantified in kidney fat, MGA is also able to be quantified in the liver of the same animal. Since the implementation of the liver method, four additional animals that showed a positive result for MGA in kidney fat also had liver tissue available for testing. Screening results from these animals (B1–B4) again show that in each case, the positive values are within the analytical range of both methods, and no values exceed the MRL. The concentrations for all points shown in Figure 7 are given in Table S4. We have also analyzed several liver samples that were negative for gestagens in kidney fat, and these samples were also negative using the new liver method. Although additional incurred sample data is needed, thus far the promising results shown in Figure 7 demonstrate the utility of using the new liver method with LC-FAIMS-SRM as a confirmatory method for the analysis of gestagens.

### 3.4 Implications and Future Work

The results from this study show that while the full method for gestagens in kidney fat using SPE with additional clean-up steps is effective, it can be replaced by a shortened method, which eliminates SPE and many clean-up steps. Although the shortened method typically has slightly more chemical background in the LC-SRM traces, the three analytes still have estimated LODs all below 0.2 ng/g, and the method readily achieves the target LLOQ of 5 ng/g. Thus, the shortened method is preferential because of the large time and cost savings in the sample preparation. In particular, the ∼6.5 h sample preparation time is shortened to less than half the time (∼2.5 h), and with the removal of the SPE cartridges, the cost is reduced to about half the cost of the full method. Since implementing the shortened kidney fat method, we have run hundreds of samples without the need for any additional instrument upkeep.

A new confirmatory method was also developed for liver tissue, which is similar to the kidney fat method in that the SPE and clean-up steps were not used. The liver extraction is simpler than commonly used QuEChERS approaches^30^ in that it is a salt-assisted liquid extraction requiring no water to be added during the extraction process, and no dispersive SPE step is needed. However, this method produced significant chemical background in the quantitative traces for the 0.6 ng/g LLOQ sample when using LC-SRM. Because of the chemical background, estimated LOD values ranged from 0.14 for MA to 0.25 ng/g for CMA, which were not sufficient. The use of a gas-phase ion separation technique (FAIMS) was used to provide additional selectivity. The positioning of the HESI probe closer to the FAIMS inlet produced more intense, partially solvated analyte ions that have different CV values compared with positioning the probe further away where complete desolvation can occur. However, once experimental conditions are fixed, the optimum CV values remain consistent, as was illustrated by peak areas for injections over 18 h. Although the signal was reduced by half with FAIMS (even more for CMA), the substantial chemical background reduction of ∼50 to 1000 times with the use of LC-FAIMS-SRM greatly improved S/N of the three gestagens and lowered estimated LODs to 0.0010–0.020 ng/g, which are a large improvement over LC-SRM LODs. The improved S/N was able to clearly show the presence of a very small MGA impurity in MGA-d_3_. Although the use of LC-FAIMS-SRM is not required in the analysis of gestagens in kidney fat in Canada since the LLOQ (5 ng/g) was readily quantifiable, the large enhancement in S/N values would assist in determining the lowest possible gestagen amounts in bovine tissues tested in the jurisdictions where lower concentrations may be of interest, for example in Europe, where the use of progestogens is banned in food animals.

Kidney fat samples with incurred MGA residues demonstrated the utility of the shortened kidney fat method as samples showed comparable levels of MGA using either the full or shortened method with LC-SRM. The analysis using the new liver method showed that MGA incurred samples in kidney fat were also positive for MGA in liver, and that kidney fat samples negative for MGA were also negative for MGA in liver, illustrating the utility of the new liver method using LC-FAIMS-SRM as a confirmatory method. These fast and effective analytical methods for the analysis of progestogens in kidney fat and liver are now being used at the CFIA for diagnostic analyses.

## Supporting Information Available

Back-calculated recovery and intermediate precision values determined during the validation of the shortened kidney fat method; quantitative method performance results for the three gestagens using three different methods for kidney fat; quantitative method performance results for the three gestagens using the new liver method with LC-FAIMS-SRM; incurred values for MGA in liver using the new liver method, and in kidney fat using the shortened kidney fat method, for the same animal; and peak areas for the quantitative traces of the three gestagens using LC-FAIMS-SRM with a HESI probe at position 1.3 for 175 injections of a 6 ng/g matrix-fortified standard (∼18 h) (PDF)
