# Supplementary material for: Simplified Liquid Chromatography–Mass Spectrometry Methods for Gestagen Analysis in Animal Fat and Liver
Source: J Agric Food Chem. 2023 Jun 15;71(25):9877–85. doi: 10.1021/acs.jafc.3c01200 (PMC10311522; doi:10.1021/acs.jafc.3c01200)

Simplified LC-MS methods for gestagen analysis in animal fat and liver

Randy W. Purves[1,2,*], Michelle West[1], Ratnadipsinh Vaghela[1], Jana Kinar[1], Yash Patel[1], Michael W. Belford[3], Bryn O. Shurmer[1]

[1]Centre for Veterinary Drug Residues, Canadian Food Inspection Agency, Saskatoon, SK S7N 2R3, Canada

[2]College of Pharmacy and Nutrition, University of Saskatchewan, Saskatoon, SK S7N 5E5, Canada

[3]Thermo Fisher Scientific, San Jose, California, 95134, United States

*Corresponding author

Email: randy.purves@usask.ca; randy.purves@inspection.gc.ca, Tel: +1 306-385-7843

Supplementary Table S1. Back-calculated recovery and intermediate precision values determined during the validation of the shortened kidney fat method.

Back-calculated recovery

MA Concentration (ng/g)	%Recovery
5 (n=13)	117.4%
10 (n=14)	111.5%
20 (n=13)	110.0%

CMA Concentration (ng/g)	%Recovery
5 (n=13)	112.2%
10 (n=14)	114.7%
20 (n=13)	113.2%

MGA Concentration (ng/g)	%Recovery
5 (n=13)	109.5%
10 (n=14)	102.8%
20 (n=13)	101.2%

Intermediate precision

MA Concentration (ng/g)	%RSD
5 (n=13)	14.1%
10 (n=14)	13.0%
20 (n=13)	12.0%

CMA Concentration (ng/g)	%RSD
5 (n=13)	16.1%
10 (n=14)	13.7%
20 (n=13)	12.5%

MGA Concentration (ng/g)	%RSD
5 (n=13)	19.0%
10 (n=14)	6.9%
20 (n=13)	6.3%

Supplementary Table S2. Quantitative method performance results for the three gestagens using three different methods for kidney fat. Matrix samples are matrix fortified.

(A) Melengestrol acetate (MGA)

**Full fat LC-SRM**				**Shortened fat LC-SRM**				**Shortened fat LC-FAIMS-SRM**			
Chem Curve (ng/g)	Value (ng/g)	% Rec	% Dev	Chem Curve (ng/g)	Value (ng/g)	% Rec	% Dev	Chem Curve (ng/g)	Value (ng/g)	% Rec	% Dev
5	5.12	102.4	2.4	5	5.09	101.8	1.8	5	5.09	101.8	1.8
	5.04	100.8	0.8		5.03	100.6	0.6		5.14	102.8	2.8
10	9.65	96.5	-3.5	10	9.9	99.0	-1.0	10	9.64	96.4	-3.6
	9.55	95.5	-4.5		9.79	97.9	-2.1		9.85	98.5	-1.5
20	20.71	103.6	3.6	20	20.1	100.5	0.5	20	19.81	99.1	-1.0
	20.60	103.0	3.0		19.96	99.8	-0.2		20.11	100.6	0.5
40	39.76	99.4	-0.6	40	40.03	100.1	0.1	40	40.77	101.9	1.9
	39.58	99.0	-1.1		40.1	100.3	0.3		39.59	99.0	-1.0
Matrix QC (ng/g)	Value (ng/g)	% Rec	% Dev	Matrix QC (ng/g)	Value (ng/g)	% Rec	% Dev	Matrix QC (ng/g)	Value (ng/g)	% Rec	% Dev
5	4.80	96.0	-4.0	5	4.93	98.6	-1.4	5	4.83	96.6	-3.4
10	9.20	92.0	-8.0	10	9.05	90.5	-9.5	10	9.10	91.0	-9.0
20	19.36	96.8	-3.2	20	19.94	99.7	-0.3	20	19.62	98.1	-1.9
Y=0.04098+0.1096*X R2=0.9987				Y = 0.02487+0.1080*X R^2 = 0.9999				Y = -0.01050+0.1135*X R^2 = 0.9994			

(B) Megestrol acetate (MA)

**Full fat LC-SRM**				**Shortened fat LC-SRM**				**Shortened fat LC-FAIMS-SRM**			
Chem Curve (ng/g)	Value (ng/g)	% Rec	% Dev	Chem Curve (ng/g)	Value (ng/g)	% Rec	% Dev	Chem Curve (ng/g)	Value (ng/g)	% Rec	% Dev
5	5.12	102.4	2.4	5	5.06	101.2	1.2	5	4.93	98.6	-1.4
	5.10	102.0	2.0		5.05	101.0	1.0		5.22	104.4	4.4
10	9.57	95.7	-4.3	10	9.88	98.8	-1.2	10	9.69	96.9	-3.1
	9.51	95.1	-4.9		9.84	98.4	-1.6		9.92	99.2	-0.8
20	20.68	103.4	3.4	20	20.05	100.3	0.3	20	20.26	101.3	1.3
	20.57	102.9	2.9		20.09	100.5	0.4		19.89	99.5	-0.5
40	40.17	100.4	0.4	40	40.02	100.1	0.1	40	40.65	101.6	1.6
	39.28	98.2	-1.8		40.02	100.1	0.1		39.45	98.6	-1.4
Matrix QC (ng/g)	Value (ng/g)	% Rec	% Dev	Matrix QC (ng/g)	Value (ng/g)	% Rec	% Dev	Matrix QC (ng/g)	Value (ng/g)	% Rec	% Dev
5	4.48	89.6	-10.4	5	4.83	96.6	-3.4	5	5.28	105.6	5.6
10	8.47	84.7	-15.3	10	9.28	92.8	-7.2	10	9.78	97.8	-2.2
20	18.03	90.2	-9.8	20	19.55	97.8	-2.3	20	21.52	107.6	7.6
Y = 0.04272+0.1017*X R^2 = 0.9985				Y = 0.02164+0.09932*X R^2 = 0.9999				Y = -0.002209+0.09836*X R^2 = 0.9993			

(C) Chlormadinone acetate (CMA)

Full fat LC-SRM				Shortened fat LC-SRM				Shortened fat LC-FAIMS-SRM			
Chem Curve (ng/g)	Value (ng/g)	% Rec	% Dev	Chem Curve (ng/g)	Value (ng/g)	% Rec	% Dev	Chem Curve (ng/g)	Value (ng/g)	% Rec	% Dev
5	5.11	102.2	2.2	5	5.04	100.8	0.8	5	4.73	94.6	-5.4
	5.27	105.4	5.4		5.01	100.2	0.2		5.35	107.0	7.0
10	9.8	98.0	-2.0	10	9.94	99.4	-0.6	10	9.59	95.9	-4.1
	9.58	95.8	-4.2		9.82	98.2	-1.8		10.17	101.7	1.7
20	20.78	103.9	3.9	20	20.18	100.9	0.9	20	19.69	98.5	-1.5
	20.59	103.0	3.0		20.18	100.9	0.9		20.49	102.5	2.4
40	39.96	99.9	-0.1	40	39.89	99.7	-0.3	40	39.07	97.7	-2.3
	39.22	98.1	-2.0		39.93	99.8	-0.2		40.92	102.3	2.3
Matrix QC (ng/g)	Value (ng/g)	% Rec	% Dev	Matrix QC (ng/g)	Value (ng/g)	% Rec	% Dev	Matrix QC (ng/g)	Value (ng/g)	% Rec	% Dev
5	5.27	105.4	5.4	5	5.04	100.8	0.8	5	4.82	96.4	-3.6
10	9.65	96.5	-3.5	10	9.56	95.6	-4.4	10	9.3	93.0	-7.0
20	20.37	101.9	1.9	20	20.13	100.7	0.6	20	19.04	95.2	-4.8

Y = 0.01212+0.04887*X R^2 = 0.9986   Y = 0.007195+0.04577*X R^2 = 0.9999   Y = -6.9314E-4+0.032665*X R^2 = 0.9982

Supplementary Table S3. Quantitative method performance results for the three gestagens using the new liver method with LC-FAIMS-SRM. Matrix samples are matrix fortified.

MGA				MA				CMA			
Matrix Curve (ng/g)	Value (ng/g)	% Rec	% Dev	Matrix Curve (ng/g)	Value (ng/g)	% Rec	% Dev	Matrix Curve(ng/g)	Value (ng/g)	% Rec	% Dev
0.6	0.61	101.3	1.3	0.6	0.61	100.8	0.8	0.6	0.62	103.0	3.0
3	3.05	101.6	1.6	3	3.12	104.0	4.0	3	2.95	98.2	-1.8
6	5.84	97.3	-2.7	6	5.61	93.6	-6.4	6	5.83	97.2	-2.8
9	8.60	95.5	-4.5	9	8.79	97.7	-2.3	9	8.98	99.7	-0.3
12	12.51	104.2	4.2	12	12.47	103.9	3.9	12	12.23	101.9	1.9
Matrix QC (ng/g)	Value (ng/g)	% Rec	% Dev	Matrix QC (ng/g)	Value (ng/g)	% Rec	% Dev	Matrix QC (ng/g)	Value (ng/g)	% Rec	% Dev
6	5.86	97.7	-2.3	6	6.27	104.5	4.5	6	6.05	100.8	0.8

Y = -0.003825+0.1922*X R^2 = 0.9978   Y = 0.001933+0.1538*X R^2 = 0.9974   Y = -0.0004969+0.05964*X R^2 = 0.9995

Supplementary Table S4. Incurred values for MGA in liver using the new liver method, and in kidney fat using the shortened kidney fat method, from the same animal.

Animal	MGA in liver (ng/g)	MGA in fat (ng/g)
A1	1.23	5.72
A1	1.26	5.74
A1	1.17	-
Avg.	1.22 ± 0.05	5.73 ± 0.01
A2	3.48	12.34
A2	3.78	12.17
A2	3.64	-
Avg.	3.64 ± 0.15	12.26 ± 0.12
A3	2.00	7.12
A3	1.99	7.06
A3	2.11	-
Avg.	2.03 ± 0.07	7.09 ± 0.04
A4	1.27	7.34
A4	1.23	7.26
A4	1.28	-
Avg.	1.26 ± 0.02	7.30 ± 0.06
A5	2.44	7.52
A5	2.85	7.45
A5	2.75	-
Avg.	2.68 ± 0.22	7.49 ± 0.05
B1	2.10	6.82
B2	1.57	8.50
B3	1.36	5.49
B4	1.30	6.55

Supplementary Figure 1. Peak areas for the quantitative traces of the three gestagens using LC-FAIMS-SRM with a HESI probe at position 1.3 for 175 injections of a 6 ng/g matrix fortified standard (~ 18 hours).

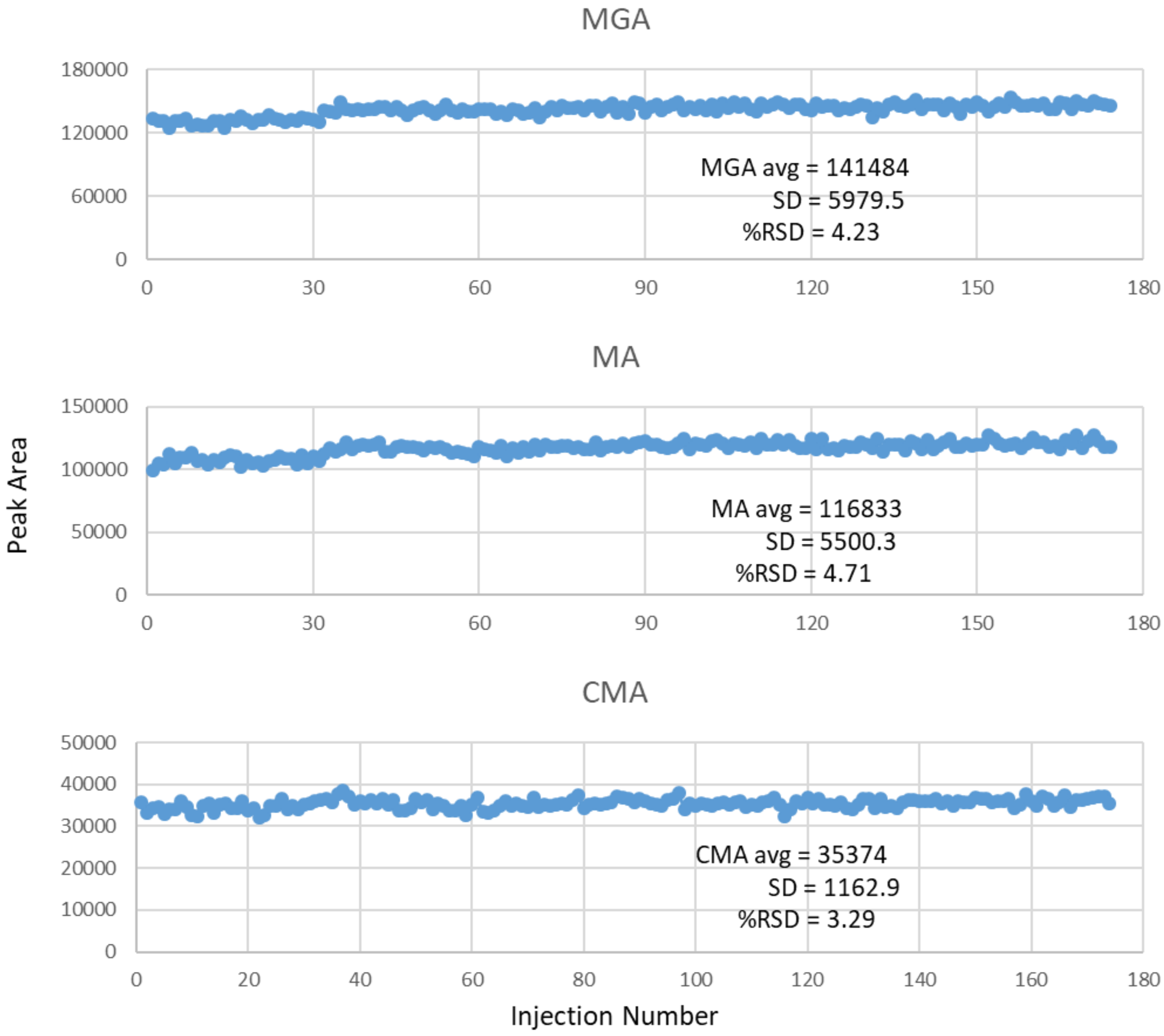

Supplement: Supplementary file 1 — jf3c01200_si_001.pdf [file jf3c01200_si_001.pdf]
